# The Philadelphia Academy of Surgery

**Published:** 1885-12

**Authors:** 


### PHILADELPHIA ACADEMY OF SURGERY.

A meeting of the Philadelphia Academy of Surgery was held October 5, 1885, Vice-President Dr. R. J. Levis in the chair.

### FOREIGN BODY CAUSING VESICAL CALCULUS.

DR. J EWTNG MEARS: I desire to present to the Academy a urinary calculus removed from a patient in St. Mary's Hospital three weeks ago. The patient was a man from the interior of the State, fifty-six years of age, who had been suffering with bladder-trouble for nine months. There had been difficult micturition with pain, and the diagnosis of inflammation of the bladder had been made. Six months ago, in order to relieve the difficulty in passing water, he said that he had introduced a straw some two or three inches long. He was under the influence of liquor at the time, and the straw slipped from his grasp and entered the urethra. His symptoms then became more marked, and he came to this city. I introduced a sound, and discovered in the bladder the stone or mass which you see. The urine was carefully examined, and it was found to contain a large quantity of albumen and also phosphatic deposits. The question arose, in view of the man's habits, his age, and the condition of the urine, whether it would be better to perform lithotomy or lithotrity or litholaplaxy. Under the circumstances, I considered lithotomy the preferable operation.

I cut the man, and in so doing opened an abscess in the prostate, evacuating about an ounce of pus. I then entered the bladder and removed this cluster of calculi with a scoop. The bladder was then washed out, and in two weeks the man returned to his home with the wound entirely closed. At the end of this time, examination of the urine showed that its character was greatly improved.

It was certainly fortunate that section of the perineum was decided upon. The abscess was not recognized before operating, although exploration of the perineum was made. There was no pain, no swelling, and no tenderness.

The question arises, in such cases as this, where the age of the man, his habits, and the composition of the urine indicate serious vesical and possibly renal disease, whether it is better to perform lithotomy or the crushing operation.

DR. LEVIS: How would the introduction of a straw account for this fimbriated character of the mass? If a head of wheat or barley had been passed, it might explain it.

DR. S. W. GROSS: Close examination will show that this is a spear of some grain, and that these little calculi are formed around the hairs of the grain.

EXCISION OF THE SCAPULA.

DR. JOHN BRINTON exhibited a specimen of interest from the magnitude of the operation required for its removal. This was a sarcoma of large size, requiring the extirpation of the entire scapula in a child eleven years old. The operation was unsuccessful as far as the life of the child was concerned, as the patient sank rapidly from the shock, and died an hour after the termination of the operation.

The tumor was fifteen inches in circumference at its base. From the history given it seemed to have originated in a fall.

The child was suffering greatly. The pain during the day-time was paroxysmal, but at night almost continuous. The following is Dr. Brinton's description of his method of operating: "I made an incision, carrying it to the posterior edge of the scapula. The incision was not at first carried the entire length, because I wished to divide the acromio-clavicular articulation as soon as possible. The idea was to save every drop of blood that could possibly be saved. I had gathered from the reports of cases that the great peril was from hemorrhage. I therefore commenced with a moderate incision, so as to divide the acromio-clavicular articulation. The incision was then swept across to the posterior portion of the bone. An incision was next made at right angles, and the incision (somewhat curved) was carried below the angle of the scapula and the four flaps dissected up. Then I commenced at the upper part of the bone, dividing the muscles ; and then passed slowly down, dividing the muscles,—taking the precaution, where there was any chance of considerable hemorrhage, to include the mass of muscle within a ligature before dividing it. Where there was no danger, a mass of tissue was grasped between two large forceps, such as I formerly used for the extraction of bullets. The incision was then carried along the posterior border and the muscles divided; it was next carried under the inferior angle of the bone and the parts raised. The incision upon the anterior costa of the scapula was carried up, the vessels being compressed; and thus the parts being lifted, I opened the capsular ligaments and turned out the head of the humerus. I next divided the heads of the muscle attached to the coracoid process. I had already divided the muscles along the spine. The bone was then readily lifted up and the hemorrhage was comparatively small. Performing the operation in this way, only one or two vessels required ligature after removal of the bone."

The microscopical examination of this growth by DR. LONGSTRETH shows that it is a well-marked example of round-celled sarcoma.

One other case of complete excision of the scapula was performed by Professor Agnew some years ago. The patient died in a short time from shock. Two partial operations were performed by the late Professor GROSS.

DR. R. J. LEVIS described a method devised by him for a similar case, by which a rubber bandage was made to surround the shoulder and control the hemorrhage.

DR. BRINTON stated that there was no difficulty in compressing the subclavian artery with the fingers in this case.

### CONGENITAL MALFORMATION OF COLON.

DR. CHARLES B. NANCREDE: I have here specimens of some little interest. They are the terminal part of the rectum and the caput coli, which were removed from an infant, 50 hours old. The child had been delivered with instruments, and seemed to be in perfect health until the second night, when the nurse sent for me and said that there was something wrong; that the child was crying and straining, but had not soiled any napkins.

On examination, I found a well-formed anus, into which I could introduce my finger one-third of an inch. It was a female child, and I could therefore make a thorough examination; but I could detect no bulging at any point. As it was twelve o'clock at night and the distention was not great, I gave an opiate, and the next morning at eleven o'clock DR. ASHHURST met me in consultation. Neither of us could feel any bowel, but we thought it right to make an effort to reach the bowel. I dissected along the hollow of the sacrum up to the promontory, but could not feel the gut. We then decided to perform the operation in the right inguinal region, and I opened what I supposed to be the sigmoid flexure; but it proved at the post mortem to be the caput coli. As soon as the peritoneal cavity was opened, about an ounce of serum escaped, and with it the right Fallopian tube, which was intensely congested. There was marked peritonitis. The child lived four and a half days after the operation. The meconium passed freely, and afterwards the discharges were natural. The child died from exhaustion, evidently due to the peritonitis.

The post mortem showed that if I had detected the bulging bowel, which I must have felt, as it was near the end of my incision, I should almost inevitably cut through two layers of peritoneum. There was a space about as wide as a director where the bowel was not covered by peritoneum, and I should have left behind the peritonitis. The question arises in these cases, if peritonitis does set in so early, and if death results, as it usually does, from peritonitis, whether it is worth while to add the double danger of two operations, especially in female children, where it is impossible to detect any sign of the bowel.

### TREATMENT OF CARBUNCLE.

DR. JAMES COLLINS: I have lately treated two cases of carbuncle on the back of the neck, by a method which seems to have some advantages. The patient is put under the influence of an anæsthetic and a linear incision made. I then take a scoop and remove all the necrosed tissue, and wash the parts thoroughly with an antiseptic solution of mercuric chloride. I then put in a drainage tube, and insert two stitches to bring the central part together. Each day the cavity is thoroughly washed out with the antiseptic solution. The patients have done well, and the cicatrix has been less than after any other method I have tried. The success depends upon the removal of the necrosed tissue and the use of the antiseptic solution.

DR. S. W. GROSS: The plan of Dr. Collins is, I think, based upon proper principles. I consider it far the best operation yet suggested. By scraping away all the dead tissue he gets rid of the micrococci which produce putrefaction, which give rise to the sloughs. The application of the corrosive sublimate destroys the micrococci which line the walls of the cavity, and in that way removes the cause of the disease.

NITROUS OXIDE GAS IN THE EXAMINATION OF FRACTURES.

J. M. BARTON, M. D., read a report of numerous cases ot fractures examined while the patient was under nitrous oxide gas. One case of re-fracture of a radius to correct a faulty union was also detailed.

The agent was found to afford sufficient relaxation and freedom from pain to enable one to diagnose and adjust most fractures. Its advantages in all minor operations, of course, are familiar. That it does not cause nausea nor vomiting, even if the stomach be not empty, the slight risk, the immediate recovery permitting the patient to attend at once to his usual avocations, etc., are well known; but in fractures we avoid that period of excitement which appears during the administration of ether, and during which the patient is so likely to further injure the fractured limb.

The period of full anæsthesia is from one to two minutes, but the period of total muscular relaxation is nearly four minutes.

While the anæsthetic is being administered, the injured limb is fully exposed and held by the surgeon. Before the patient is quite unconscious the surgeon feels the limb become limp and lax in his hand; all the muscles are relaxed. The examination can now begin, though the patient gives some slight evidence of feeling pain. This period, the period of total unconsciousness, and the succeeding period of muscular relaxation, gives about four minutes, which will be found to be abundant time to examine almost any fracture.

The apparatus for administering nitrous oxide is now both cheaper and more portable than formerly. The dental depots supply the liquified gas in small receivers, thus doing way with the necessity of tanks and large rubber bags. Its use in surgery will probably increase, notwithstanding that it is slightly more expensive than chloroform or ether.

